# DJ-1 is dispensable for human stem cell homeostasis

**DOI:** 10.1007/s13238-019-00659-9

**Published:** 2019-10-22

**Authors:** Fang Cheng, Si Wang, Moshi Song, Zunpeng Liu, Ping Liu, Lei Wang, Yanjiang Wang, Qian Zhao, Kaowen Yan, Piu Chan, Weiqi Zhang, Jing Qu, Guang-Hui Liu

**Affiliations:** 1 National Laboratory of Biomacromolecules, CAS Center for Excellence in Biomacromolecules, Institute of Biophysics, Chinese Academy of Sciences, Beijing, 100101 China; 2 State Key Laboratory of Membrane Biology, Institute of Zoology, Chinese Academy of Sciences, Beijing, 100101 China; 3 State Key Laboratory of Stem Cell and Reproductive Biology, Institute of Zoology, Chinese Academy of Sciences, Beijing, 100101 China; 4 Advanced Innovation Center for Human Brain Protection, National Clinical Research Center for Geriatric Disorders, Xuanwu Hospital Capital Medical University, Beijing, 100053 China; 5 Department of Neurology, Daping Hospital, Third Military Medical University, Chongqing, 400042 China; 6 CAS Key Laboratory of Genomic and Precision Medicine, Beijing Institute of Genomics, Chinese Academy of Sciences, Beijing, 100101 China; 7 University of Chinese Academy of Sciences, Beijing, 100049 China; 8 Institute for Stem cell and Regeneration, Chinese Academy of Sciences, Beijing, 100101 China; 9 Translational Medical Center for Stem Cell Therapy, Shanghai East Hospital, Tongji University School of Medicine, Shanghai, 200120 China

**Dear Editor,**

Safeguarding cellular redox homeostasis is crucial for maintaining organism health and preventing diseases. Oxidative stress, often characterized by decreased mitochondrial integrity and increased reactive oxygen species (ROS) production, disrupts proteostasis and genomic stability, which may eventually lead to cellular decomposition (Oh et al., 2014). Stem cells (such as mesenchymal stem cells, MSCs, and neural stem cells, NSCs) are susceptible to various external and internal stresses, and their dysfunction contributes to aging and aging-related diseases. Thus, it is of importance to elucidate the complex signaling networks regulated by oxidative stress in stem cells. Although redox signaling has been implicated in multiple cellular processes, how the redox system functions in various human stem cells is unclear.

DJ-1 is considered to be a key regulator of cellular redox homeostasis. It was first identified as an oncogene and later a causative gene for autosomal recessive early-onset Parkinson’s disease (Bonifati et al., 2003; Nagakubo et al., 1997). Later, DJ-1 was reported to sense and protect against oxidative stress in neuronal cells (Biosa et al., 2017; Taira et al., 2004; Zhang et al., 2018). As an antioxidant protein, DJ-1 not only eliminates peroxide under oxidative stress by auto-oxidation but also regulates the transcription of *NRF2* and its target genes (Biosa et al., 2017; Clements et al., 2006). Recently, DJ-1 was reported to function as a deglycase against lipid peroxidation, DNA oxidation and glucose oxidation implicated in aging and neurodegenerative disorders (Richarme et al., 2017; Sharma et al., 2019). While a number of studies have revealed the critical roles of DJ-1 in regulating the cellular oxidative state in multiple somatic cell lines and animal models (neuronal cells, human tumor cell lines, drosophila and mice) (Biosa et al., 2017; Kahle et al., 2009; Taira et al., 2004), the biological function of DJ-1 in human stem cells remains largely unknown.

To investigate the role of DJ-1 in various human diploid cells, especially in human stem cells, we first generated DJ-1 knockout human embryonic stem cells (hESCs) using the CRISPR/Cas9 technique (Figs. 1A and S1A). Loss of DJ-1 protein was confirmed by Western blotting and immunofluorescence staining (Fig. 1B and 1C). The use of both N-terminal and C-terminal antibodies demonstrated that DJ-1 was completely ablated in *DJ-1*^−/−^ hESCs. Immunofluorescence staining revealed that DJ-1 was localized mainly in the cytoplasm but also in the nuclei of wild type (WT, *DJ-1*^+/+^) hESCs, and was absent in *DJ-1*^−/−^ hESCs (Fig. 1C). The *DJ-1*^−/−^ hESCs expressed pluripotency markers (Figs. 1D and S1B), were able to differentiate into all three germ layer lineages (Fig. S1C), and maintained normal karyotype (Fig. S1D). In addition, no remarkable difference in cell proliferation ability, cell cycle kinetics, or ROS levels was observed between *DJ-1*^−/−^ and WT hESCs (Figs. 1E, 1F, S1E and S1F). These observations indicate that DJ-1 is dispensable for the maintenance of hESC self-renewal and pluripotency.

**Figure 1:**
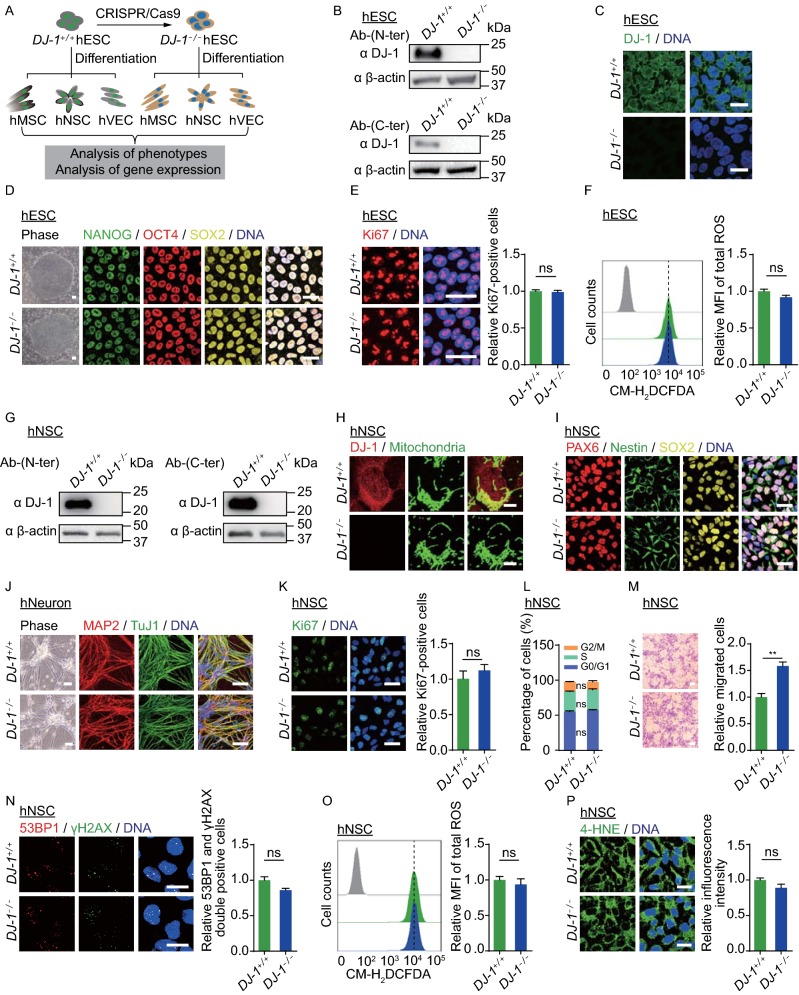
**DJ-1 deficiency exhibited a minimal impact on hESCs and hNSCs**. (A) Schematic diagram of the generation of *DJ-1*^−/−^ hESCs, as well as the generation of hMSCs, hNSCs and hVECs. (B) Western blotting analysis of DJ-1 expression in hESCs using anti-DJ-1 antibodies (N-terminus and C-terminus). β-actin was used as the loading control. (C) Immunofluorescence analysis of DJ-1 expression in WT and *DJ-1*^−/−^ hESCs. Scale bar, 25 µm. (D) Phase-contrast images of hESCs are shown to the left. Scale bar, 50 µm. Immunofluorescence staining of the pluripotency markers is shown to the right. Scale bar, 25 µm. (E) Immunofluorescence analysis of Ki67 expression in WT and *DJ-1*^−/−^ hESCs. Scale bar, 25 µm. Data are presented as the mean ± SEM, *n* = 3. ns, not significant. (F) Flow cytometry analysis of total ROS levels in WT and *DJ-1*^−/−^ hESCs. Data are presented as the mean ± SEM, *n* = 3. ns, not significant. MFI, median fluorescence intensity. (G) Western blotting analysis of DJ-1 expression in hNSCs using anti-DJ-1 antibodies (N-terminus and C-terminus). β-actin was used as the loading control. (H) Immunofluorescence analysis of DJ-1 expression in WT and *DJ-1*^−/−^ hNSCs. Scale bar, 7.5 µm. (I) Immunofluorescence analysis of hNSC markers in WT and *DJ-1*^−/−^ hNSCs. Scale bar, 25 µm. (J) Phase-contrast images of hNeurons to the left. Scale bar, 50 µm. Immunofluorescence staining of hNeuron**-**specific markers in WT and *DJ-1*^−/−^ hNeurons to the right. Scale bar, 25 µm. (K) Immunofluorescence analysis of Ki67 expression in WT and *DJ-1*^−/−^ hNSCs. Scale bar, 25 µm. Data are presented as the mean ± SEM, *n* = 3. ns, not significant. (L) Cell cycle analysis of WT and *DJ-1*^−/−^ hNSCs. Data are presented as the mean ± SEM, *n* = 3. ns, not significant. (M) Migration abilities of WT and *DJ-1*^−/−^ hNSCs were evaluated by Transwell assay. Data are shown as the mean ± SEM, *n* = 3. Scale bar, 50 µm. ** *P* < 0.01. (N) Immunofluorescence analysis of 53BP1 and γH2AX expression in WT and *DJ-1*^−/−^ hNSCs. Data are shown as the mean ± SEM, *n* = 3. ns, not significant. Scale bar, 25 µm. (O) Cellular total ROS levels were determined by staining with the CM-H_2_DCFDA probe and quantified by FACS. Data are presented as the mean ± SEM, *n* = 3. ns, not significant. (P) Immunofluorescence analysis of 4-HNE expression in WT and *DJ-1*^−/−^ hNSCs. Data are shown as the mean ± SEM, *n* = 3. ns, not significant. Scale bar, 25 µm

To investigate the role of DJ-1 in human neural stem cells (hNSCs), we directly differentiated WT and *DJ-1*^−/−^ hESCs into hNSCs. Ablation of DJ-1 was verified by Western blotting in *DJ-1*^−/−^ hNSCs (Fig. 1G). Immunofluorescence staining revealed that DJ-1 was mainly localized in the cytoplasm and partially in the nuclei and mitochondria in WT hNSCs (Fig. 1H). Both WT and *DJ-1*^−/−^ hNSCs expressed typical hNSC markers, including PAX6, Nestin and SOX2 (Fig. 1I). Importantly, *DJ-1*^−/−^ hNSCs were able to efficiently differentiate into human neurons (hNeurons) as did WT hNSCs (Figs. 1J and S1G), indicating that DJ-1 deficiency had no adverse effect on neuronal differentiation ability of hNSCs. In addition, Ki67 immunofluorescence staining, clonal formation, and cell cycle analysis showed comparable proliferation abilities between WT and *DJ-1*^−/−^ hNSCs (Figs. 1K,1L and S1H). By comparison, enhanced cell migration was observed in *DJ-1*^−/−^ hNSCs (Fig. 1M). The expression of DNA damage response markers 53BP1 and γH2AX showed no difference between WT and *DJ-1*^−/−^ hNSCs (Fig. 1N). We next sought to explore whether the absence of DJ-1 resulted in oxidative stress and mitochondrial dysfunction in hNSCs. No difference was detected in cellular ROS levels, lipid peroxidation, or mitochondrial mass between WT and *DJ-1*^−/−^ hNSCs (Figs. 1O, 1P and S1I). Because DJ-1 has been implicated in cellular response to oxidative and mitochondrial stress, cell viabilities of WT and *DJ-1*^−/−^ hNSCs were examined upon treatment with various oxidative and mitochondrial stress inducers (PX-12, paraquat, carbonyl cyanide 3-chlorophenylhydrazone (CCCP), and thenoyltrifluoroacetone (TTFA)) and no marked difference was detected between WT and *DJ-1*^−/−^ hNSCs (Fig. S1J). Likewise, no difference in cell viability was observed between WT and *DJ-1*^−/−^ hNSCs in response to other types of cellular toxins, including DNA damage inducers (Zeocin, mitomycin C (MMC), camptothecin (CPT)) and a proteasomal inhibitor (MG132) (Fig. S1K and S1L). Taken together, our results indicate that DJ-1 deficiency minimally affects hNSC homeostasis.

We next differentiated WT and *DJ-1*^−/−^ hESCs into human mesenchymal stem cells (hMSCs). Both WT and *DJ-1*^−/−^ hMSCs were positive for hMSC markers, including CD73, CD90, and CD105 (Fig. S2A), and negative for hMSC-irrelevant markers, such as CD34, CD43 and CD45 (Fig. S2B). Western blotting analysis confirmed the loss of DJ-1 in *DJ-1*^−/−^ hMSCs (Fig. S2C). DJ-1 was predominantly localized in the cytoplasm and partially in the mitochondria in WT hMSCs (Fig. 2A). We observed comparable differentiation abilities towards osteoblasts, chondrocytes and adipocytes between WT and *DJ-1*^−/−^ hMSCs (Fig. S2D). Proliferation ability assessed by Ki67 immunostaining (Fig. 2B), senescence-associated β-galactosidase (SA-β-gal) positivity (Fig. 2C), and DNA damage response assessed by 53BP1 and γH2AX immunostaining and comet assays showed no remarkable difference between WT and *DJ-1*^−/−^ hMSCs (Figs. 2D and S2E). In addition, *DJ-1*^−/−^ hMSCs displayed comparable cellular redox levels by 4-HNE staining, CM-H_2_DCFDA probe, MitoSOX Red probe, NADPH sensor iNap1, and glutathione sensor roGFP targeted to the cytosol and endoplasmic reticulum (ER) as those in WT hMSCs (Figs. 2E, 2F and S2F–H), indicating that DJ-1 deficiency did not disrupt redox homeostasis in hMSCs. Mitochondrial mass levels were also comparable between WT and *DJ-1*^−/−^ hMSCs (Fig. 2G). In contrast to the recently identified function of DJ-1 as a deglycase (Richarme et al., 2017), we did not observe marked difference between WT and *DJ-1*^−/−^ hMSCs in ER stress responses to high glucose (Fig. S2I), or cell viabilities to glycation stress inducer (methylglyoxal, MGO) (Fig. S2J) or ER stress inducer (tunicamycin, TM) (Fig. S2K). Cell viabilities of WT and *DJ-1*^−/−^ hMSCs were further evaluated upon treatment with oxidative stress inducers (PX-12, H_2_O_2_, TTFA) and DNA damage inducers (MMC, Zeocin, 4-nitroquinoline N-oxide (4NQO), CPT) but no remarkable difference was detected (Fig. S2L and S2M). Additionally, we injected WT and *DJ-1*^−/−^ hMSCs transduced with a lentiviral vector expressing luciferase (Luc) into the tibialis anterior (TA) muscles of immunodeficient mice and observed comparable hMSC decay rates (Fig. 2H), indicating that DJ-1 deficiency did not accelerate hMSC attrition *in vivo.*

**Figure 2:**
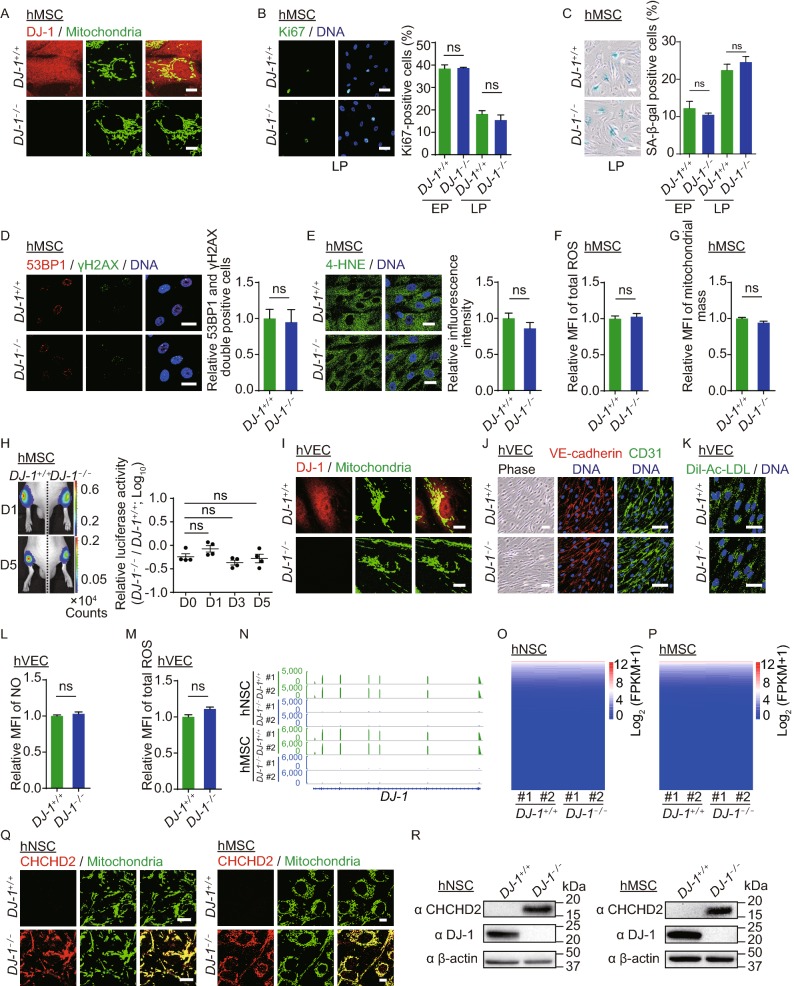

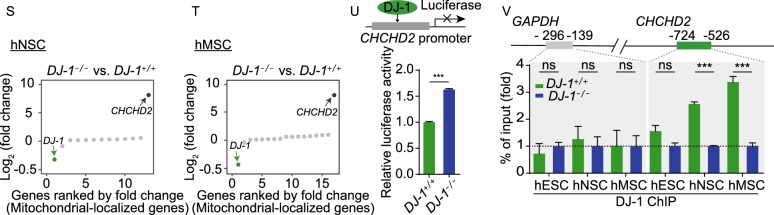
**DJ-1-deficient hMSCs and hVECs maintained cellular homeostasis**. (A) Immunofluorescence analysis of DJ-1 expression in WT and *DJ-1*^−/−^ hMSCs. Scale bar, 10 µm. (B) Immunofluorescence analysis of Ki67 expression in WT and *DJ-1*^−/−^ hMSCs. Left, images of immunostaining for the Ki67 at P8 (LP, late passage). Right, quantification of Ki67-positive cells in WT and *DJ-1*^−/−^ hMSCs at P4 (EP, early passage) and P8 (LP). Scale bar, 25 µm. Data are presented as the mean ± SEM, *n =* 3. ns, not significant. (C) SA-β-gal staining of WT and *DJ-1*^−/−^ hMSCs. Left, images of SA-β-gal staining at P9 (LP, late passage). Right, quantification of SA-β-gal-positive cells in WT and *DJ-1*^−/−^ hMSCs at P4 (EP) and P9 (LP). Scale bar, 50 μm. Data are presented as the mean ± SEM, *n* = 3. ns, not significant. (D) Immunofluorescence analysis of 53BP1 and γH2AX expression in WT and *DJ-1*^*−/−*^ hMSCs. Data are presented as the mean ± SEM, *n* = 3. ns, not significant. Scale bar, 25 μm. (E) Immunofluorescence analysis of 4-HNE expression in WT and *DJ-1*^−/−^ hMSCs. Data are shown as the mean ± SEM, *n* = 3. ns, not significant. Scale bar, 25 µm. (F) Cellular total ROS levels were determined by staining with the CM-H_2_DCFDA probe and analyzed by FACS. Data are presented as the mean ± SEM, *n* = 3. ns, not significant. (G) Mitochondrial mass levels were determined by staining with NAO probe and measured by FACS. Data are presented as the mean ± SEM, *n* = 3. ns, not significant. (H) Analysis of luciferase activity in the TA muscles of immunodeficient mice by an *in vivo* imaging system (IVIS). WT (1 × 10^6^, left) and *DJ-1*^−/−^ (1 × 10^6^, right) hMSCs (passage 6) transduced with luciferase were implanted into the muscles of mice. Luciferase activities were imaged and quantified at days 0, 1, 3, and 5 after implantation. Data are presented as the mean ± SEM, *n* = 4. ns, not significant. (I) Immunofluorescence analysis of DJ-1 expression in WT and *DJ-1*^−/−^ hVECs. Scale bar, 10 μm. (J) Phase-contrast images of hVECs to the left. Scale bar, 50 µm. Immunofluorescence staining of hVEC-specific markers, VE-cadherin and CD31 to the right. Scale bar, 25 μm. (K) Immunofluorescence staining of Dil-Ac-LDL in WT and *DJ-1*^−/−^ hVECs. Scale bar, 25 µm. (L) Flow cytometry analysis of nitric oxide (NO) levels in WT and *DJ-1*^−/−^ hVECs. Data are presented as the mean ± SEM, *n* = 3. ns, not significant. (M) Flow cytometry analysis of total ROS levels in WT and *DJ-1*^−/−^ hVECs. Data are presented as the mean ± SEM, *n* = 3. ns, not significant. (N) Transcriptional signals of *DJ-1* in WT and *DJ-1*^−/−^ hNSCs and hMSCs. Data were normalized by RPKM at bin size of 10 bp. (O) Heatmap illustrating FPKM normalized expression level of each gene in WT and *DJ-1*^−/−^ hNSCs. (P) Heatmap illustrating FPKM normalized expression level of each gene in WT and *DJ-1*^−/−^ hMSCs. (Q) Immunofluorescence staining of CHCHD2 in WT and *DJ-1*^−/−^ hNSCs (left) and hMSCs (right). Scale bar, 10 µm. (R) Western blotting analysis of CHCHD2 and DJ-1 expression in hNSCs (left), and hMSCs (right). β-actin was used as the loading control. (S) Scatter plot showing the fold change of mitochondrial-localized genes (adjusted *P* ≤ 0.05) in *DJ-1*^−/−^ hNSCs compared to WT hNSCs. (T) Scatter plot showing the fold change of mitochondrial-localized genes (adjusted *P* ≤ 0.05) in *DJ-1*^−/−^ hMSCs compared to WT hMSCs. (U) Transcriptional activity of *CHCHD2* in WT and *DJ-1*^−/−^ hMSCs measured by dual luciferase reporter assay. WT and *DJ-1*^−/−^ hMSCs were co-transfected with pGL3-*CHCHD2* promoter and Renilla plasmids. Data are presented as the mean ± SEM, *n* = 3. ***P < 0.001. (V) ChIP-qPCR assessment of the enrichment of DJ-1 at the *CHCHD2* promoter in hESCs, hNSCs and hMSCs. Data are presented as the mean ± SEM, *n* = 4. ***P < 0.001, ns, not significant

We next investigated whether DJ-1 regulates the homeostasis of human vascular endothelial cells (hVECs) by differentiating WT and *DJ-1*^−/−^ hESCs into hVECs. Loss of DJ-1 protein in hVECs was confirmed by Western blotting and immunostaining (Figs. 2I and S3A). DJ-1 was localized mainly in the cytoplasm and nucleus and partially in the mitochondria of WT hVECs (Fig. 2I). Both WT and *DJ-1*^−/−^ hVECs expressed vascular endothelial markers, including VE-cadherin and CD31 (Fig. 2J). Clonal formation and cell cycle analysis showed comparable proliferation abilities between WT and *DJ-1*^−/−^ hVECs (Fig. S3B and S3C). Acetylated low density lipoprotein (Dil-Ac-LDL) uptake (Fig. 2K), nitric oxide (NO) synthesis (Fig. 2L), and cell migration (Fig. S3D) showed no difference between WT and *DJ-1*^−/−^ hVECs. Similar to the observations made in hNSCs and hMSCs, *DJ-1*^−/−^ hVECs exhibited comparable ROS levels to those of WT hVECs (Fig. 2M), suggesting that DJ-1 was dispensable for the redox homeostatic maintenance of hVECs.

Because DJ-1 is a transcriptional co-activator (Biosa et al., 2017), we next performed RNA sequencing in WT and *DJ-1*^−/−^ hNSCs and hMSCs (Figs. 2N–P and S3E–L). Notably, we observed minimal global changes in the gene expression profile of *DJ-1*^−/−^ hNSCs and hMSCs compared with those of WT hNSCs and hMSCs, respectively (Fig. 2O and 2P). While DJ-1 has been reported to regulate the transcription of *NRF2* and its target genes (Biosa et al., 2017), we did not detect any marked change in the expression levels of *NRF2* target genes between WT and *DJ-1*^−/−^ hNSCs and hMSCs (Fig. S3G and S3H). Only 12 upregulated genes and 18 downregulated genes were identified in *DJ-1*^−/−^ hNSCs, and 37 upregulated genes and 33 downregulated genes were found in *DJ-1*^−/−^ hMSCs relative to their WT counterparts (Fig. S3I and S3J). Venn diagram analysis revealed 3 commonly upregulated genes and 3 commonly downregulated genes upon DJ-1 deficiency in hMSCs and hNSCs (Fig. S3K). Among those genes, we found that *CHCHD2,* a mitochondrial-localized antioxidant gene, was upregulated in both *DJ-1*^−/−^ hNSCs and hMSCs (Fig. S3L) (Aras et al., 2015). The expression levels of CHCHD2 were upregulated in *DJ-1*^−/−^ hNSCs, hMSCs, hNeurons and hVECs, but not in *DJ-1*^−/−^ hESCs, revealed by RT-qPCR, immunofluorescence and Western blotting (Figs. 2Q, 2R, S3M and S3N). Other mitochondrial-localized genes were not differentially regulated by DJ-1 deficiency in hNSCs and hMSCs (Figs. 2S, 2T, S3O and S3P), suggesting the possible transcriptional regulation of *CHCHD2* by DJ-1. We next cloned the *CHCHD2* promoter upstream of a luciferase reporter and detected increased *CHCHD2* promoter activity in *DJ-1*^−/−^ hMSCs compared with that of WT controls (Fig. 2U). We further observed that DJ-1 bound to *CHCHD2* promoter in WT hNSCs and hMSCs, but not in WT hESCs (Fig. 2V), suggesting that DJ-1 may transrepress *CHCHD2* transcription in non-pluripotent cells by binding to the promoter of *CHCHD2*. Additionally, increased levels of active histone mark H3K4me3 and decreased levels of repressive histone mark H3K27me3 at *CHCHD2* promoter were detected in *DJ-1*^−/−^ hNSCs (Fig. S3Q and S3R). These results are in line with the notion that DJ-1 functions as a transcriptional repressor of *CHCHD2* in hNSCs and hMSCs.

In this study, we generated DJ-1-deficient hESCs and differentiated them into human adult stem cells, including hNSCs and hMSCs, as well as hVECs, providing valuable experimental models for studying the biological roles of DJ-1 in various human cell types. Consistent with previous studies (Kahle et al., 2009), DJ-1 was mainly localized in the cytoplasm and nucleus and partially in the mitochondria of all cell types tested. Although DJ-1 has been implicated in antioxidative pathways and deglycation (Richarme et al., 2017; Taira et al., 2004), we found that DJ-1 deletion had no adverse effect on hESCs, hNSCs, hMSCs and hVECs at baseline and even upon treatment with various stress inducers. In particular, the absence of oxidative stress hallmarks in those *DJ-1*^−/−^ cells suggests that DJ-1 may be dispensable in the maintenance of redox homeostasis of human stem cells, at least those we tested. In addition, the absence of DJ-1 exhibited minimal impact on global gene expression. We did not observe any difference in the expression of genes previously reported to be regulated by DJ-1 in some human tumor cell lines and experimental animal models (rat, mouse, fly), such as *NRF2* and its target genes (Biosa et al., 2017; Kahle et al., 2009). It is therefore possible that the antioxidative and transcription-regulatory functions of DJ-1 were species- or cell-type-specific. Interestingly, *CHCHD2* transcription was remarkably upregulated in DJ-1-deficient hNSCs, hMSCs, hNeurons and hVECs, but not in DJ-1-deficient hESCs. We showed, for the first time, that DJ-1 could act as a transcriptional repressor, as binding of DJ-1 to the *CHCHD2* promoter is associated with the silencing of CHCHD2 in human adult stem cells, but not in hESCs that often exhibit strong buffering capability to various cellular defects (Zhang et al., 2013). Given that DJ-1 has been reported to act as a transcriptional regulator for *NRF2* and *P53* (Biosa et al., 2017; Clements et al., 2006), DJ-1 might transrepress *CHCHD2* expression by regulating certain client transcription factors, which awaits further investigation. As CHCHD2 is a mitochondrial-localized antioxidative protein whose deficiency disrupts mitochondrial integrity (Aras et al., 2015), the increased CHCHD2 expression we observed might reflect a compensatory antioxidative response to DJ-1 deficiency that contributed to the maintenance of mitochondrial integrity and cellular redox homeostasis. Interestingly, increased CHCHD2 expression has been linked to enhanced migration of human neurons (Shimojima et al., 2015), which is consistent with the enhanced cell migration in DJ-1-deficient hNSCs and provides a plausible clue for understanding the early pathogenesis of Parkinson’s disease. However, no difference was detected in the migration abilities between WT and *DJ-1*^−/−^ hMSCs (data not shown) and hVECs, suggesting that the regulation of DJ-1 on cell migration may be cell lineage-specific. Recent studies causatively link *CHCHD2* mutations with autosomal dominant and sporadic Parkinson’s disease (Funayama et al., 2015), whereas loss-of-function mutations of *DJ-1* have been associated with autosomal recessive Parkinson’s disease with unknown mechanisms (Bonifati et al., 2003). Therefore, the normal interplay between DJ-1 and CHCHD2 may be required for the maintenance of functional homeostasis of human dopaminergic neurons, whose deregulation contributes to the onset of Parkinson’s disease (Damier et al., 1999). Based on our results that CHCHD2 was upregulated in DJ-1-deficient hNSCs, pan-neurons, hMSCs and hVECs, we speculated that the expression of CHCHD2 may be deregulated in DJ-1-deficient dopaminergic neurons. Yet, the interplay between DJ-1 and CHCHD2 in dopaminergic neurons is still unclear, which warrants further investigation.

In summary, our data showed that the absence of DJ-1 had no adverse effect on proliferation, differentiation, and oxidative stress responses of human stem cells, such as hNSCs and hMSCs as well as hVECs, suggesting that loss of DJ-1 function alone is insufficient to disrupt the homeostasis of these human cells. In addition, we found that CHCHD2 was upregulated upon DJ-1 deficiency, which may account for the absence of severe phenotypes in various types of DJ-1-deficient cells. For the first time, our study revealed the ‘see-saw’ expression pattern of two Parkinson’s disease-associated genes, providing potential clues for understanding the mechanisms of *DJ-1*- and *CHCHD2*-associated Parkinson’s disease.

## Electronic supplementary material

Below is the link to the electronic supplementary material.

Supplementary material 1 (PDF 1801 kb)
