# Correction to: DJ-1 is dispensable for human stem cell homeostasis

**DOI:** 10.1093/procel/pwaf034

**Published:** 2025-05-20

This is a correction to: Fang Cheng, Si Wang, Moshi Song, Zunpeng Liu, Ping Liu, Lei Wang, Yanjiang Wang, Qian Zhao, Kaowen Yan, Piu Chan, Weiqi Zhang, Jing Qu, Guang-Hui Liu, DJ-1 is dispensable for human stem cell homeostasis, *Protein & Cell*, Volume 10, Issue 11, November 2019, Pages 846–853, https://doi.org/10.1007/s13238-019-00659-9

In the original publication, in Fig. 1 panel I, the immunofluorescence images for PAX6 in DJ-1^+/+^ and DJ-1^-/-^ group were incorrectly labeled. The images for DJ-1^+/+^ and DJ-1^-/-^ were swapped. The corrected image is as follows:


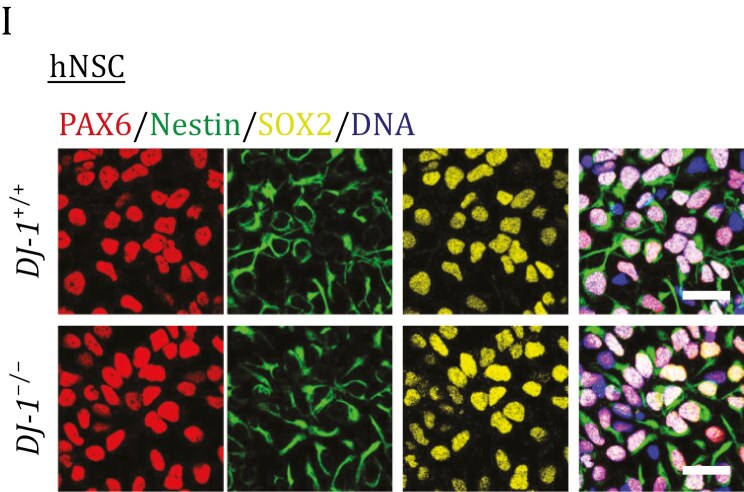


The correction does not alter the overall conclusions of the study and the corrected images support the findings presented in the original article.

These details have been corrected only in this correction notice to preserve the published version of record.

