# Supplementary material for: DJ-1 is dispensable for human stem cell homeostasis
Source: Protein Cell. 2019 Oct 22;10(11):846–53. doi: 10.1007/s13238-019-00659-9 (PMC6834734; doi:10.1007/s13238-019-00659-9)
Supplement: Supplementary file 1 — Supplementary material 1 (PDF 1801 kb) [file 13238_2019_659_MOESM1_ESM.pdf]

Supplemental Materials

Materials and Methods

Cell culture

Wild-type hESCs (Line H9, female, WiCell Research) and derived *DJ-1*$^{-/-}$ hESCs were maintained on Mitomycin C (MMC) (Selleck)-inactivated mouse embryonic fibroblast (MEF) feeders in DMEM/F12 (Gibco) medium supplemented with 20% Knockout Serum Replacement (Gibco), 0.1 mM non-essential amino acids (NEAA, Gibco), 2 mM GlutaMAX (Gibco), 1% penicillin/streptomycin (Gibco), 55 μM β-mercaptoethanol (Thermo Fisher Scientific), and 10 ng/ml FGF2 (Joint Protein Central, JPC) or on Matrigel (Corning) in mTeSR medium (STEMCELL Technologies). hMSCs were maintained on gelatin (Sigma)-coated plates (Corning) in α-MEM medium (Gibco) containing 10% fetal bovine serum (FBS, Gibco), 0.1 mM NEAA (Gibco), 1% penicillin/streptomycin (Gibco), and 1 ng/mL FGF2 (JPC). hNSCs were cultured in neural stem cell maintenance medium (NSMM) supplemented with 50% Neurobasal (Gibco), 50% Advanced DMEM/F12 (Gibco), 2 mM GlutaMAX, 0.1 mM NEAA (Gibco), 1% penicillin/streptomycin (Gibco), 1×B27 (Gibco), 1×N2 (Gibco), 10 ng/mL human leukemia inhibitory factor (hLIF, Millipore), 2 μM SB431542 (Selleck) and 3 μM CHIR99021 (Selleck). hVECs were cultured on collagen (Advanced BioMatrix)-coated plates in EGM-2 (Lonza) medium containing 50 ng/mL VEGF-165 (HumanZyme), 20 ng/mL FGF2 (JPC) and 10 nM SB431542 (Selleck).

Generation of DJ-1 knockout (*DJ-1*$^{-/-}$) hESCs

The DJ-1 protein was ablated using the CRISPR/Cas9-mediated gene-editing technique. In brief, gRNA targeting exon 2 of the *DJ-1* gene was cloned into the gRNA vector (Addgene #41824). H9 ESCs were pretreated with the ROCK inhibitor Y-27632 (Selleck) for 24 hr before plasmid electroporation. The H9 ESCs were dissociated with TrypLE (Thermo Fisher Scientific), and 5×10^6 H9 ESCs were electroporated with 7 μg pCAGmCherry-gRNA (Addgene #87110) vectors and 14 μg pCAG-1BPNLS-Cas9-1BPNLS-2AGFP (Addgene #87109) vectors. After 48 hr, mCherry and GFP double-positive cells were isolated by FACS (BD, Aria II) and then cultured on MMC-inactivated MEF cells in hESC culture medium. After approximately two weeks in culture, emerging colonies expanded**,** and then, each single clone was picked into a 24-well culture plate and expanded for identification. The sequence for the *DJ-1* guide RNA (gRNA) was 5'-GAGATGTCATGAGGCGAGCT-3'. The primers for clone identification were 5'-TGTGAAATGCAGGAGACCCACGTA-3' (forward) and 5'-TGAGCGACACAGAGAGACACCATC-3' (reverse).

Generation and characterization of hNSCs

Differentiation of hESCs into hNSCs was described previously (Wang et al., 2019). In brief, hESCs were cultured on MEF feeders in hESC medium for approximately two days and then in NID-I medium containing 50% Neurobasal (Gibco), 50% Advanced DMEM/F12 (Gibco), 2 mM GlutaMAX (Gibco), 0.1 mM NEAA (Gibco), 1% penicillin/streptomycin (Gibco), 1×B27 (Gibco), 1×N2 (Gibco), 10 ng/mL hLIF (Millipore), 3 μM SB431542 (Selleck), 4 μM CHIR99021 (Selleck), 2 μM dorsomorphin (Sigma) and 0.1 μM Compound E (EMD Chemicals, Inc.). Two days later, the NID-I medium was replaced by NID-II medium containing 50% Neurobasal,

50% Advanced DMEM/F12, 2 mM GlutaMAX, 0.1 mM NEAA (Gibco), 1% penicillin/streptomycin (Gibco), 1×B27, 1×N2, 10 ng/mL hLIF, 3 μM SB431542 and 4 μM CHIR99021 and 0.1 μM Compound E for another five days. Then, the hNSCs were passaged on Matrigel-coated plates in neural stem cell maintenance medium (NSMM) containing 50% Neurobasal, 50% Advanced DMEM/F12, 1×B27, 1×N2, 2 mM GlutaMAX, 0.1 mM NEAA, 1% penicillin/streptomycin, 10 ng/mL hLIF, 2 μM SB431542 and 3 μM CHIR99021.

Neuronal differentiation

hNSCs were seeded at a density of 3×10^4 cells per well in a Matrigel-coated 24-well plates and cultured in neural stem cell maintenance medium for 1-3 days. The cells were then cultured in differentiation medium containing DMEM/F12, 1×N2, 1×B27, 200 μM Ascorbic acid (Sigma), 400 μM dbcAMP (Sigma), 10 ng/mL GDNF (Peprotech) and 10 ng/mL of BDNF (Peprotech) for two days before 20 μg/mL laminin (Sigma) was added to further promote differentiation. After 14 days, differentiated hNeurons were subjected to immunostaining with neuronal markers MAP2 and TuJ1 and Western blotting analysis.

Generation and characterization of hMSCs

Differentiation of hESCs into hMSCs was performed as described previously (Wang et al., 2019). Briefly, embryoid bodies (EBs) were plated on Matrigel-coated plates in hMSC differentiation medium containing 90% α-MEM, 10% FBS (Gibco), 0.1 mM NEAA (Gibco), 1% penicillin/streptomycin (Gibco), 10 ng/mL FGF2 (JPC) and 5 ng/mL TGFβ (HumanZyme). Approximately 10 days later, the fibroblast-like cells appeared. The cells were than passaged on gelatin-coated plates in hMSC culture medium supplemented with 90% α-MEM (Gibco), 10% fetal bovine serum (FBS, Gibco), 0.1 mM NEAA (Gibco), 1% penicillin/streptomycin (Gibco), and 1 ng/mL FGF2 (JPC). CD73, CD90 and CD105 tri-positive cells were then sorted as hMSCs by FACS (BD, Aria II). Identification of hMSC-specific markers with antibodies including anti-human CD73-PE (BD, 550257), anti-human CD90-FITC (BD, 555595) and anti-human CD105-APC (BD, 323208). Negative hMSC markers CD34, CD43 and CD45 were also analyzed with antibodies including anti-human CD34-PE (BD, 555822), anti-human CD43-APC (BD, 560198) and anti-human CD45-FITC (BD, 555482) by an LSR Fortessa cell analyzer (BD). To evaluate the multiple-lineage differentiation capacities, hMSCs were differentiated towards chondrocytes, osteoblasts, and adipocytes as previously described (Wang et al., 2019). Toluidine blue O (Sigma, 89640, chondrogenesis), Von Kossa (Genmed Scientifics, GMS 80045.3, osteogenesis), and Oil red O (Sigma, O1391, adipogenesis) staining analyses were performed.

Generation and characterization of hVECs

Differentiation of hESCs into hVECs was described previously (Wu et al., 2018). Briefly, hESCs were first cultured with M1 medium, including 25 ng/mL BMP4 (R&D), 3 μM CHIR99021 (Selleck), 3 μM IWP2 (Selleck) and 4 ng/mL FGF2 (JPC). After three days, the M1 medium was replaced by M2 medium containing 50 ng/mL VEGF-165 (HumanZyme), 20 ng/mL FGF2 (JPC), and 10 ng/mL IL-6 (Peprotech). At Day 6, differentiated cells were harvested using Accumax (Millipore) and stained with anti-human CD144-APC (BD, 561567) and anti-human CD201-PE (BD, 351904) antibodies and hVECs were sorted by a flow cytometer (BD, Aria II).

Flow cytometry analysis

For cell cycle analysis, cells were fixed with 70% precooled ethanol at least overnight and then treated with staining buffer containing 0.1% Triton X-100 (Sigma), 0.2 mg/mL RNase A (Sangon Biotech) and 0.02 mg/mL propidium iodide (Molecular Probes) at 37 ℃ for 30 min. Cellular total ROS, mitochondrial ROS, mitochondrial mass and nitric oxide levels were measured using CM-H_2DCFDA (Thermo Fisher Scientific), MitoSOX Red (Thermo Fisher Scientific), nonyl acridine orange (NAO, Thermo Fisher Scientific) and DAF-FM (Thermo Fisher Scientific) according to the manufacturer's protocols. Cells were then analyzed by an LSR Fortessa cell analyzer (BD). The cell cycle data were analyzed by ModFit software.

SA-β-Gal staining assay

SA-β-Gal staining was performed as described previously (Wu et al., 2018). Briefly, cells were fixed in fixation buffer containing 2% (w/v) formaldehyde and 0.2% (w/v) glutaraldehyde for 5 min at room temperature. After fixation, cells were stained with staining buffer containing 1 mg/mL X-gal overnight at 37 ℃. Stained cells were observed by optical microscopy, and the percentages of positive cells were quantified by ImageJ software.

Clonal expansion assay

A total of 2000 cells were seeded in each well of a 12-well plate (Corning) and cultured for approximately two weeks. For hESCs, 10 μM Y-27632 (Selleck) was added on the first day of cell passaging and removed after 24 hr. The relative colony number and relative cell integral density were calculated by ImageJ software after crystal violet staining.

Western blotting

To detect the expression of DJ-1 and CHCHD2, cells were lysed in 2×SDS-sample buffer and boiled at 105 ℃ for 10 min. To detect the unfolded protein response of ER, cells were lysed in lysis buffer (Millipore) with protease inhibitor cocktail (Roche) and phosphatase inhibitor cocktail (Roche). A following BCA kit (bicinchoninic acid) was used for protein quantification. Generally, 20 μg of cell lysate was loaded onto SDS-PAGE gels for electrophoresis and electrotransferred to PVDF membranes (Millipore). After blocking with 5% (w/v) nonfat powdered milk (BBI Life Sciences), the membrane was incubated with primary antibodies overnight at 4 ℃ and HRP-conjugated secondary antibodies followed at room temperature for 1 hr. Finally, imaging was performed with the ChemiDoc XRS system (Bio-Rad). The primary antibodies used for Western blotting in this study were anti-DJ-1 (N-terminus) (Abcam, ab76008), anti-DJ-1 (C-terminus) (Abcam, ab4150), anti-β-actin (Santa Cruz, sc-69879), anti-CHCHD2 (Proteintech, 19424-1-AP), anti-eIF2α (Cell Signaling Technology, 5324), anti-Phospho-eIF2α (Cell Signaling Technology, 9721), anti-IRE1α (Cell Signaling Technology, 3294), anti-IRE1 (Abcam, ab124945), anti-PERK (Cell Signaling Technology, #3192), anti-GAPDH (Sigma, G8795), and anti-BiP (Sigma, G8918).

Immunofluorescence

Cells were fixed with 4% formaldehyde at room temperature for 30 min, permeabilized with 0.4% Triton X-100 in PBS at room temperature for 30 min and blocked with 10% donkey serum (Jackson ImmunoResearch Labs) in PBS at room

temperature for 1 hr. The cells were then incubated with primary antibodies at 4 ℃ overnight followed by incubation with corresponding fluorescent secondary antibodies at room temperature for 1 hr and counterstained with Hoechst 33342 (Thermo Fisher Scientific). Cell images were taken using a confocal microscope (Leica SP5). The primary antibodies used were as follows (company, catalogue number): anti-DJ-1 (Abcam, ab76008), anti-NANOG (Abcam, ab21624), anti-OCT3/4 (Santa Cruz, sc-5279), anti-SOX2 (Santa Cruz, sc-17320), anti-Ki67 (ZSGB-Bio, ZM-0166), anti-PAX6 (Biolegend, 901301), anti-Nestin (BD, 560422), anti-MAP2 (Sigma, M4403), anti-β-tubulin III (TuJ1, Sigma, T2200), anti-SMA (Abcam, 32575), anti-FOXA2 (Cell Signaling Technology, 8186S), anti-VE-cadherin (Cell Signaling Technology, 2158S), anti-CD31 (BD, 555445), anti-γH2AX (Millipore, 05-636), anti-53BP1 (Bethyl Laboratories, A300-273A), anti-CHCHD2 (Proteintech, 19424-1-AP), anti-4 Hydroxynonenal (4-HNE, Abcam, ab46545), and anti-Mitochondria (Millipore, MAB1273).

Determination of cytosol and ER redox states

hMSCs were infected with PLE4-cytosol-roGFP or PLE4-superfolded-ro-GFP-iE_{ER} lentiviruses (Zhang et al., 2019b) for 72 hr, harvested and washed twice with Hanks' Balanced Salt Solution (HBSS, Gibco), and then seeded onto a flat-bottom 96-well plate. The fluorescence intensities were measured at 525 nm with excitation at 390 and 465 nm using an EnSpire Multimode Plate Reader (Perkin Elmer), and the fluorescence intensity ratio at 390/465 nm excitation was calculated.

Detection of intracellular NADPH levels

hMSCs were infected with PLE4-iNap1 or PLE4-iNapc lentiviruses for 72 hr, harvested and washed twice with Hanks' Balanced Salt Solution (HBSS, Gibco), and then seeded onto a flat-bottom 96-well plate. The fluorescence intensities were measured at 528 nm with excitation at 420 and 485 nm using an EnSpire Multimode Plate Reader (Perkin Elmer), and the fluorescence intensity ratio at 420/485 nm excitation was calculated. iNapc was used to correct the iNap1.

Cell viability analysis

hMSCs (1×10^4) were seeded on 0.1% gelatin-coated 96-well plates (Corning), and upon reaching 90% confluence**,** cells were treated with various stress inducers for 24 hr. Cell viability was measured using the MTS approach according to the recommended protocol for the CellTiter 96 AQueous One Solution Cell Proliferation Assay (Promega, G3582). hNSCs (2.5×10^4) were seeded on Matrigel-coated 96-well plates (Corning), and upon reaching 90% confluence**,** the cells were treated with indicated stress inducers for 24 hr. The stress inducers used in this assay includes DMSO (vehicle, Sigma, D2650), PX-12 (Santa Cruz Biotech, 358518), H_2O_2 (Sigma, 323381), TTFA (Sigma, T27006), 4NQO (Sigma, N8141), MMC (Selleck, S8146), paraquat (Sigma, 36541), zeocin (Selleck, S1214), MGO (Sigma, M0252-25), MG132 (Sigma, M7449), CPT (Sigma, C9911), TM (Sigma, 11089-65-9), CCCP (Sigma, C2759).

RNA extraction, RT-PCR, and RT-qPCR

Total RNA was extracted by TRIzol reagent (Thermo Fisher Scientific). Approximately 2 μg of total RNA was used for cDNA synthesis using the GoScript Reverse Transcription System (Promega). For RT-PCR, cDNA was used as a

template with the following primers and PrimeSTAR HS DNA polymerase (TaKaRa) followed by gel electrophoresis for semi-quantification of genes of interest.
GAPDH forward primer, 5’-GGAGCGAGATCCCTCCAAAAT-3’,
GAPDH reverse primer, 5’-GGCTGTTGTCATACTTCTCATGG-3’,
SOX2 forward primer, 5’-CAAAAATGGCCATGCAGGTT-3’,
SOX2 reverse primer, 5’-AGTTGGGATCGAACAAAAGCTATT-3’,
OCT4 forward primer, 5’-GGGTTTTTGGGATTAAGTTCTTCA-3’,
OCT4 reverse primer, 5’-GCCCCCACCCTTTGTGTT-3’,
NANOG forward primer, 5’-ACAACTGGCCGAAGAATAGCA-3’,
NANOG reverse primer, 5’-GGTTCCCAGTCGGGTTCAC-3’.

RT-qPCR was performed using THUNDERBIRD SYBR qPCR Mix (TOYOBO) on a CFX-384 Real-Time PCR system (Bio-Rad). The relative expression of genes was normalized to the *GAPDH* transcript. The qPCR primers used are as follows.
GAPDH forward primer, 5’-GGAGCGAGATCCCTCCAAAAT-3’,
GAPDH reverse primer, 5’-GGCTGTTGTCATACTTCTCATGG-3’,
CHCHD2 forward primer, 5’-ACACATTGGGTCACGCCATTA-3’,
CHCHD2 reverse primer, 5’-GCACCTCATTGAAACCCTCACA-3’.

Cell migration assay
For the wound healing assay, 2×10^4 hVECs were seeded on collagen-coated 96-well plates (Corning). At a confluence of 90%, cells were analyzed by IncuCyte S3 (ESSEN BIOSCIENCE). For the Transwell assay, 5×10^4 hNSCs were cultured on the top chamber of the Transwells (24-well insert; pore size, 8 μm; Corning) in 200 μL basal medium. Another 500 μL of complete culture medium was added into the bottom chambers. After 24 hr, cells that migrated through the Transwell were stained with crystal violet and counted by ImageJ software.

Dil-Ac-LDL uptake assay
In brief, hVECs were collected after 6-hr incubation with Dil-Ac-LDL (Molecular Probes) in hVEC culture medium. For immunofluorescence detection, cells were processed following the immunofluorescence protocol.

Luciferase reporter assay
Partial *CHCHD2* promoter (-1500 bp - 0 bp) were amplified by PCR and cloned into the pGL3-Basic vector (Promega). The pGL3-*CHCHD2* promoter plasmid together with a Renilla plasmid (Promega), which was used as an internal control, were co-transfected into hMSCs with Lipofectamine 3000 (Thermo Fisher Scientific). After two days, cells were harvested using Dual-Luciferase assay kit (Vigorous Biotechnology, Beijing, China), and firefly and Renilla luciferase activity were measured with a Synergy H1 Hybrid Reader (BioTek). The PCR primers for the sequence of *CHCHD2* promoter were shown as below: forward: 5’-CCGGGTACCATGCACCCATCACCTGAGCAA-3’, reverse: 5’-CGGCTCGAGCTGAACTTGGCGCCAGGATCT-3’.

ChIP-qPCR
ChIP-qPCR assay was performed according to a previously published protocol with slight modifications (Zhang et al., 2019a). In brief, cells were harvested and

crosslinked by 1% (v/v) formaldehyde for 14 min (DJ-1) or 10 min (H3K4me3 and H3K27me3) followed by the termination of crosslinking with 125 mmol/L glycine for 5 min. After washed with PBS, the cells were lysed and sonicated to shear the chromatin into fragments. The product was incubated with antibody-binding Protein A dynabeads overnight at 4 ℃. The supernatant was then removed and the beads were decrosslinked for 2 hr at 68 ℃. The DNA was purified by phenolchloroform-isoamylalcohol and chloroformisoamylalcohol extractions and quantified by qPCR. Antibodies for ChIP included anti-DJ-1 (Abcam, ab4150), anti-H3K4me3 (Abcam, ab8580) and anti-H3K27me3 (Abcam, ab192985). The primers for detection of the DJ-1 binding locus at the *CHCHD2* promoter were 5'-AACACATGGCTTGCTTCC-3' (forward) and 5'-ATGGAGATACTGCACCAA-3' (reverse). The *GAPDH* promoter were used as the negative control and the primers for detection were 5'-CACAGTCCAGTCCTGGGAAC-3' (forward) and 5'-TAGTAGCCGGGCCCTACTTT-3' (reverse).

Animal experiments

All animal experiments were conducted with approval of the Institute of Biophysics, Chinese Academy of Sciences. Teratoma assays were performed as described (Zhang et al., 2019a). In brief, approximately 5×10^6 hESCs were injected into the groin cavities of NOD/SCID mice (male, 4 weeks). After approximately 2 months, the teratomas were removed and analyzed by immunofluorescence staining. The hMSC transplantation assay was carried out as described previously (Wang et al., 2019). In brief, cells were transduced with lentiviruses that express luciferase. A total of 1×10^6 cells were injected into the mid-portion of the TA muscle of nude mice (male, 6-8 weeks). Mice were imaged in vivo with an IVIS spectrum imaging system (XENOGEN, Caliper) every two days.

RNA-seq library construction and sequencing

Total RNA of hMSCs (P3) or hNSCs (P5) was extracted by TRIzol (Thermo Fisher Scientific). Library construction, sequencing and processing of RNA-seq data were carried out as previously described (Wang et al., 2019). Briefly, RNA integrity was examined by the Bioanalyzer 2100 system (Agilent Technologies), and then, libraries were constructed using NEBNext Ultra™ RNA Library Prep Kit for Illumina (NEB) and sequenced on an Illumina HiSeq X Ten platform.

RNA-seq data processing

Paired-end raw reads were trimmed with Trim Galore (v0.4.5, http://www.bioinformatics.babraham.ac.uk/projects/trim_galore), and cleaned reads were mapped to the UCSC hg19 human genome using hisat2 (v2.0.4) with default parameters. Aligned reads were then annotated and counted by HTSeq (v0.6.1). FPKM (Fragment Per Kilobase per Million mapped reads) normalized expression level of each gene was computed by StringTie. Differentially expressed genes (DEGs) were analyzed using DESeq2 with a cutoff Benjamini-Hochberg adjusted *P* value (adjusted *P*) ≤ 0.05 and absolute $\log_2$(fold change) ≥ 1. The Pearson correlation coefficient (R) of RNA-seq replicates was computed based on DESeq2 regularized logarithm (rLog) normalized read counts. To visualize the RNA-seq signals at *DJ-1* and *CHCHD2* genomic loci in WT and *DJ-1*$^{-/-}$ hNSCs and hMSCs, RNA signals

were normalized by RPKM (Reads Per Kilobase Million) at 10 bp bin size by deepTools2. The RNA-seq data have been deposited to the NCBI Gene Expression Omnibus (GEO) database with accession number GSE136834.

Statistical analysis

Data are presented as the mean ± SEM. GraphPad Prism software was used to perform a two-tailed Student's *t*-test. Statistical significance is presented as * $P < 0.05$, ** $P < 0.01$ and *** $P < 0.001$.

Figure S1. Generation and functional analyses of *DJ-1*$^{-/-}$ hESCs and hNSCs.

(A) Schematic of the deletion of *DJ-1* via CRISPR/Cas9-mediated non-homologous end-joining (NHEJ). (B) RT-PCR analysis showing comparable mRNA expression levels of pluripotency markers, including *SOX2, OCT4,* and *NANOG,* between WT and *DJ-1*$^{-/-}$ hESCs. (C) Immunofluorescence staining of TuJ1 (ectoderm), SMA (mesoderm) and FOXA2 (endoderm) in teratomas derived from WT and *DJ-1*$^{-/-}$ hESCs. Scale bar, 50 μm. (D) Karyotype analysis of *DJ-1*$^{-/-}$ hESCs. (E) Clonal expansion analysis of WT and *DJ-1*$^{-/-}$ hESCs. Data are presented as the mean ± SEM, $n = 3$. ns, not significant. (F) Cell cycle analysis of WT and *DJ-1*$^{-/-}$ hESCs. Data are presented as the mean ± SEM, $n = 3$. ns, not significant. (G) Immunofluorescence analysis of DJ-1 expression in WT and *DJ-1*$^{-/-}$ hNeurons. Scale bar, 25 μm. (H) Clonal expansion analysis of WT and *DJ-1*$^{-/-}$ hNSCs. Data are presented as the mean ± SEM, $n = 3$. ns, not significant. (I) Mitochondrial mass levels were determined by staining with NAO probe and measured by FACS. Data are presented as the mean ± SEM, $n = 3$. ns, not significant. (J-L) MTS cell viability assay for WT and *DJ-1*$^{-/-}$ hNSCs treated with oxidative and mitochondrial stress inducers (PX12, paraquat, CCCP and TTFA) marked by orange color (J), DNA damage inducers (Zeocin, MMC, CPT) marked by blue color (K) and a proteasomal inhibitor (MG132) marked by green color (L). Data were normalized to the vehicle control group (Vehicle). Data are shown as the mean ± SEM, $n = 3$. ns, not significant.

Figure S2. Characterization and functional analyses of *DJ-1*$^{-/-}$ hMSCs.

(A) Flow cytometry analysis showing the expression of hMSC markers CD73, CD90, and CD105 in WT and *DJ-1*$^{-/-}$ hMSCs. (B) Flow cytometry analysis showing the absence of the non-hMSC markers CD34, CD43, and CD45 in WT and *DJ-1*$^{-/-}$ hMSCs. (C) Western blotting analysis of DJ-1 expression in hMSCs using anti-DJ-1 antibodies (N-terminus and C-terminus). β-actin was used as the loading control. (D) Osteogenesis, chondrogenesis and adipogenesis of WT and *DJ-1*$^{-/-}$ hMSCs. Von Kossa, Alcian blue and Oil Red-O were used to characterize osteoblasts, chondrocytes and adipocytes, respectively. Scale bar, 50 μm. (E) Comet assay analysis showing the DNA damage of WT and *DJ-1*$^{-/-}$ hMSCs. Data are presented as the mean ± SEM, $n = 45$ cells for each group. ns, not significant. WT hMSCs were exposed to the UV (1200 J/m^2) to induce DNA damage and was used as positive control (PC). (F) Mitochondrial ROS levels were determined by staining with MitoSOX Red probe and measured by FACS. Data are presented as the mean ± SEM, $n = 3$. ns, not significant. (G) NADPH levels in WT and *DJ-1*$^{-/-}$ hMSCs were determined by the ratio of iNap1 fluorescence excited at 420 nm and 485 nm normalized by iNapc. Data are presented as the mean ± SEM, $n = 3$. ns, not significant. (H) Glutathione redox states in the cytosol and ER of WT and *DJ-1*$^{-/-}$ hMSCs were determined by the fluorescence ratio

of roGFP and superfolded-roGFP-iE$_{ER}$ excited at 390 and 465 nm, respectively. Data are presented as the mean ± SEM, n = 3. ns, not significant. (I) Unfolded protein response of WT and *DJ-1*$^{-/-}$ hMSCs cultured in low glucose (LG, 5 mM) and high glucose (HG, 25 mM) medium were measured by Western blotting. NC, negative control. PC, positive control. (J-M) MTS cell viability for WT and *DJ-1*$^{-/-}$ hMSCs treated with indicated concentrations of glycation stress inducer (MGO) marked by pink color (J), ER stress inducer (TM) marked by yellow color (K), oxidative stress inducers (PX12, H_2O_2, TTFA) marked by orange color (L) and DNA damage inducers (MMC, Zeocin, 4NQO, CPT) marked by blue color (M). Data were normalized to the vehicle control group (Vehicle). Data are shown as the mean ± SEM, n = 3. ns, not significant.

Figure S3 Functional analyses of hVECs and mRNA expression profiling of hNSCs and hMSCs.

(A) Western blotting analysis of DJ-1 expression in hVECs using anti-DJ-1 antibodies (N-terminus and C-terminus). β-actin was used as the loading control. (B) Clonal expansion analysis of WT and *DJ-1*$^{-/-}$ hVECs. Data are presented as the mean ± SEM, n = 3. ns, not significant. (C) Cell cycle analysis of WT and *DJ-1*$^{-/-}$ hVECs. Data are presented as the mean ± SEM, n = 3. ns, not significant. (D) Cell migration analysis of WT and *DJ-1*$^{-/-}$ hVECs. Scale bar, 100 μm. Data are shown as the mean ± SEM. n = 4. ns, not significant. (E-F) Scatter plots showing the correlation between replicates of WT and *DJ-1*$^{-/-}$ hNSCs (E) and hMSCs (F). (G-H) Density plots showing $\log_2$ (fold change) of mRNA expression levels between WT and *DJ-1*$^{-/-}$ in hNSCs (G) and hMSCs (H) for *NRF2* and its target genes. (I-J) Volcano plots showing differentially expressed genes between *DJ-1*$^{-/-}$ and WT hNSCs (I) and hMSCs (J). Green indicates downregulated genes; blue indicates upregulated genes. (K) Venn diagrams showing the numbers of commonly downregulated and upregulated genes in *DJ-1*$^{-/-}$ hNSCs and hMSCs. (L) Transcriptional signals of *CHCHD2* in WT and *DJ-1*$^{-/-}$ hNSCs and hMSCs. Data were normalized by RPKM at a bin size of 10 bp. (M) RT-qPCR analysis of *CHCHD2* in hNSCs, hMSCs, hNeurons and hESCs. Data are presented as the mean ± SEM, n = 4. ***P<0.001, ns, not significant. (N) Western blotting analysis of CHCHD2 and DJ-1 expression in hNeurons, hVECs and hESCs. β-actin was used as the loading control. (O-P) Density plots showing $\log_2$ (fold change) of mRNA expression levels between *DJ-1*$^{-/-}$ and WT in hNSCs (O) and hMSCs (P) for mitochondrial-localized genes. (Q) ChIP–qPCR assessment of the enrichment of H3K4me3 at the *CHCHD2* promoter in WT and *DJ-1*$^{-/-}$ hNSCs. Data are presented as the mean ± SEM, n = 4. ***P<0.001, ns, not significant. (R) ChIP-qPCR assessment of the enrichment of H3K27me3 at the *CHCHD2* promoter in WT and *DJ-1*$^{-/-}$ hNSCs. Data are presented as the mean ± SEM, n = 4. ***P<0.001, ns, not significant.

REFERENCES

Wang, S., Min, Z., Ji, Q., Geng, L., Su, Y., Liu, Z., Hu, H., Wang, L., Zhang, W., Suzuiki, K., *et al.* (2019). Rescue of premature aging defects in Cockayne syndrome stem cells by CRISPR/Cas9-mediated gene correction. Protein & cell.

Wu, Z., Zhang, W., Song, M., Wang, W., Wei, G., Li, W., Lei, J., Huang, Y., Sang, Y., Chan, P., *et al.* (2018). Differential stem cell aging kinetics in Hutchinson-Gilford progeria syndrome and Werner syndrome. Protein & cell *9*, 333-350.
Zhang, X., Liu, Z., Liu, X., Wang, S., Zhang, Y., He, X., Sun, S., Ma, S., Shyh-Chang, N., Liu, F., *et al.* (2019a). Telomere-dependent and telomere-independent roles of RAP1 in regulating human stem cell homeostasis. Protein & cell.
Zhang, Y., Li, T., Zhang, L., Shangguan, F., Shi, G., Wu, X., Cui, Y., Wang, X., Wang, X., Liu, Y., *et al.* (2019b). Targeting the functional interplay between endoplasmic reticulum oxidoreductin-1alpha and protein disulfide isomerase suppresses the progression of cervical cancer. EBioMedicine *41*, 408-419.

Figure S1

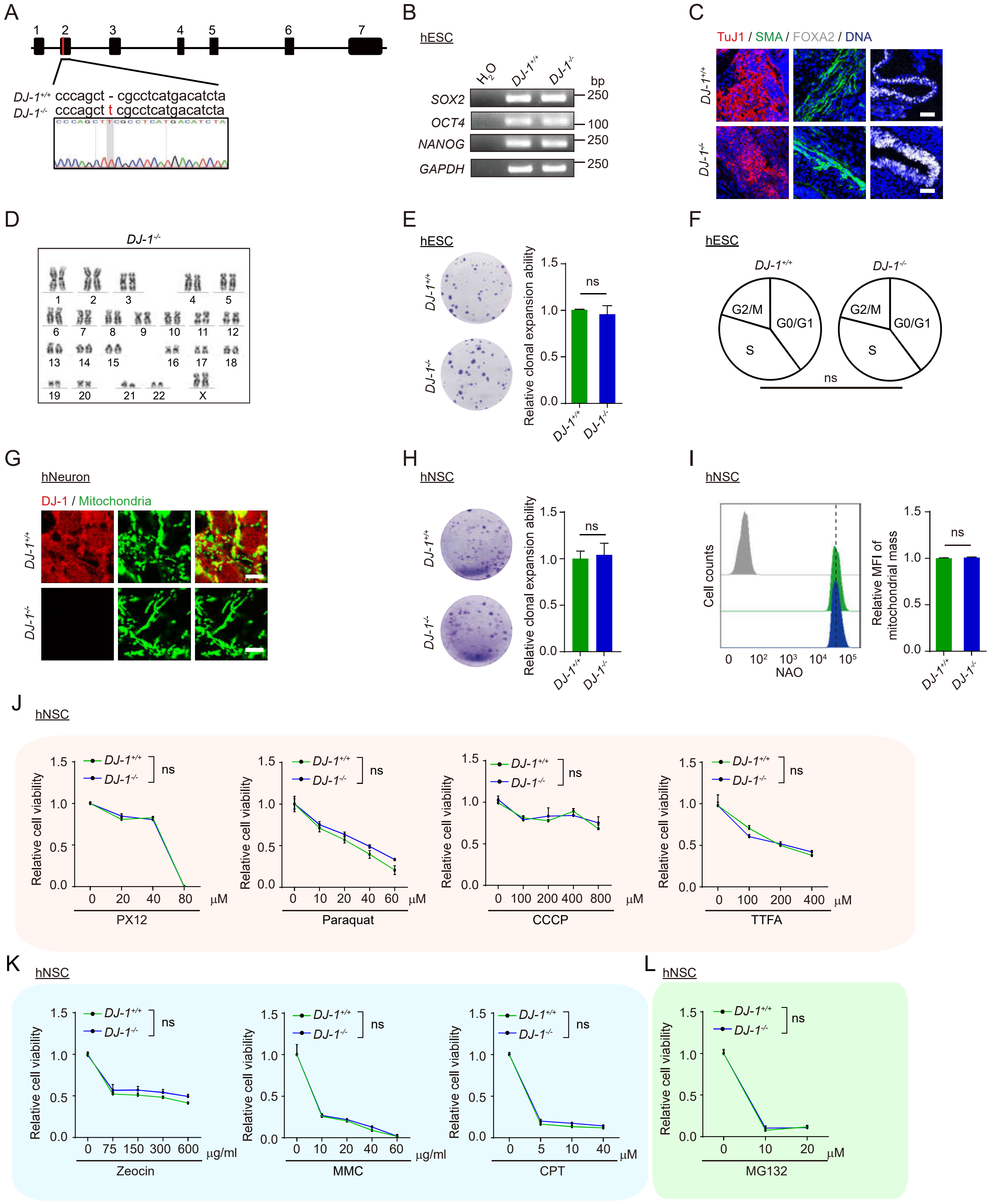
A
1 2 3 4 5 6 7
DJ-1+/+ cccagct - cgcctcatgacatcta
DJ-1-/- cccagct t cgcctcatgacatcta
B
hESC
H2O DJ-1+/+ DJ-1-/- bp
SOX2 250
OCT4 100
NANOG 250
GAPDH 250
C
TuJ1 / SMA / FOXA2 / DNA
DJ-1+/+
DJ-1-/-
D
DJ-1-/-
1 2 3 4 5
6 7 8 9 10 11 12
13 14 15 16 17 18
19 20 21 22 X
E
hESC
DJ-1+/+
DJ-1-/-
Relative clonal expansion ability
ns
F
hESC
DJ-1+/+ DJ-1-/-
G2/M G0/G1 S
ns
G
hNeuron
DJ-1 / Mitochondria
DJ-1+/+
DJ-1-/-
H
hNSC
Relative clonal expansion ability
ns
I
hNSC
Cell counts
NAO
Relative MFI of mitochondrial mass
ns
J
hNSC
Relative cell viability
DJ-1+/+
DJ-1-/-
ns
PX12 μM
Paraquat μM
CCCP μM
TTFA μM
K
hNSC
Zeocin μg/ml
MMC μg/ml
CPT μM
L
hNSC
MG132 μM

Figure S2

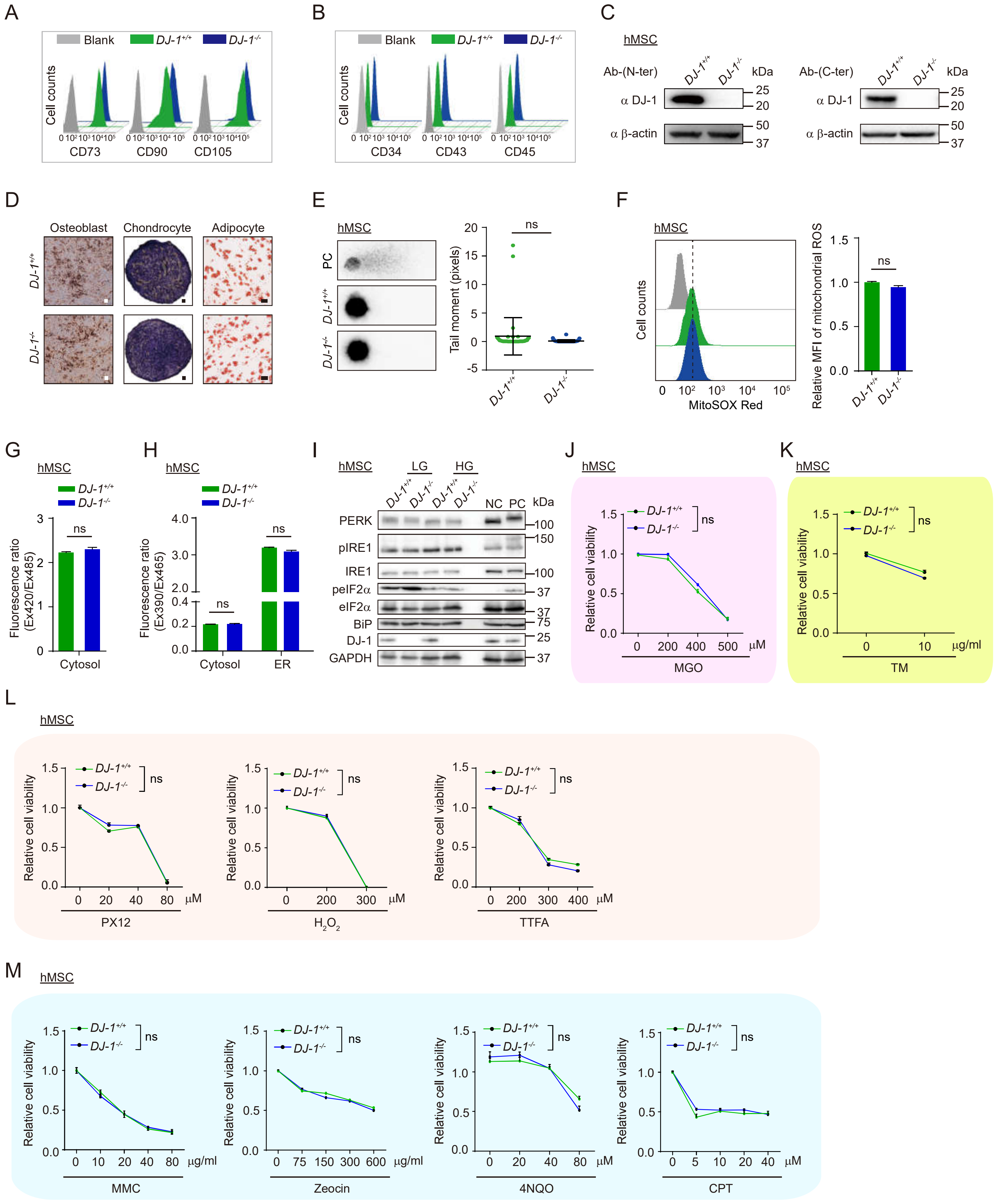

A
Blank DJ-1+/+ DJ-1-/-
Cell counts
CD73 CD90 CD105
B
CD34 CD43 CD45
C
hMSC
Ab-(N-ter) Ab-(C-ter)
α DJ-1
α β-actin
kDa 25 20 50 37
D
Osteoblast Chondrocyte Adipocyte
E
hMSC
PC
Tail moment (pixels)
ns
F
hMSC
Cell counts
MitoSOX Red
Relative MFI of mitochondrial ROS
G
hMSC
Fluorescence ratio (Ex420/Ex485)
Cytosol
H
hMSC
Fluorescence ratio (Ex390/Ex465)
Cytosol ER
I
hMSC
LG HG NC PC kDa
PERK
pIRE1
IRE1
peIF2α
eIF2α
BiP
DJ-1
GAPDH
J
hMSC
Relative cell viability
MGO
K
hMSC
TM
L
hMSC
PX12
H2O2
TTFA
M
hMSC
MMC
Zeocin
4NQO
CPT

Figure S3

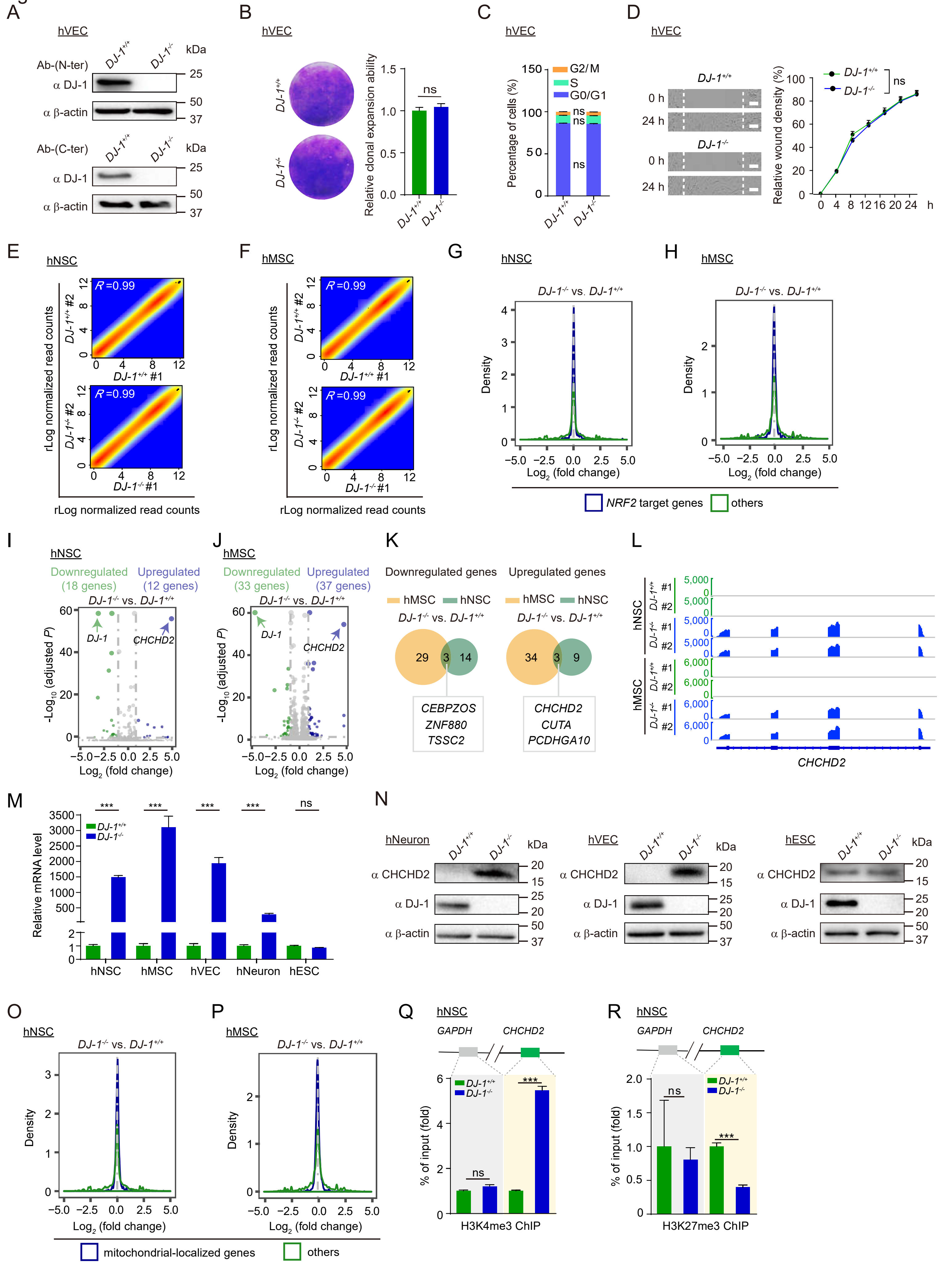

A
hVEC
Ab-(N-ter)
DJ-1+/+
DJ-1-/-
kDa
α DJ-1
α β-actin
Ab-(C-ter)
B
hVEC
Relative clonal expansion ability
ns
C
hVEC
Percentage of cells (%)
G2/M
S
G0/G1
ns
D
hVEC
0 h
24 h
Relative wound density (%)
h
E
hNSC
R=0.99
rLog normalized read counts
F
hMSC
G
hNSC
DJ-1-/- vs. DJ-1+/+
Density
Log2 (fold change)
H
hMSC
NRF2 target genes
others
I
hNSC
Downregulated (18 genes)
Upregulated (12 genes)
-Log10 (adjusted P)
DJ-1
CHCHD2
J
hMSC
Downregulated (33 genes)
Upregulated (37 genes)
K
Downregulated genes
Upregulated genes
hMSC
hNSC
29
3
14
34
9
CEBPZOS
ZNF880
TSSC2
CHCHD2
CUTA
PCDHGA10
L
hNSC
hMSC
#1
#2
5,000
6,000
CHCHD2
M
Relative mRNA level
***
ns
hNSC
hMSC
hVEC
hNeuron
hESC
N
hNeuron
hVEC
hESC
α CHCHD2
α DJ-1
α β-actin
O
hNSC
P
hMSC
mitochondrial-localized genes
others
Q
hNSC
GAPDH
CHCHD2
% of input (fold)
H3K4me3 ChIP
R
hNSC
H3K27me3 ChIP
